# Antimicrobial activity and rutin identification of honey produced by the stingless bee *Melipona compressipes manaosensis* and commercial honey

**DOI:** 10.1186/1472-6882-13-151

**Published:** 2013-07-01

**Authors:** Renah Boanerges de Queiroz Pimentel, Cristovão Alves da Costa, Patrícia Melchionna Albuquerque, Sergio Duvoisin Junior

**Affiliations:** 1 Universidade do Estado do Amazonas, Programa de Pós-graduação em Biotecnologia e Recursos Naturais da Amazônia, Av. Carvalho Leal, 1777, Manaus, AM, 69.065-170, Brazil; 2 Instituto Nacional de Pesquisas da Amazônia, Av. André Araújo, 2936, Manaus, AM, 69.060-000, Brazil; 3 Universidade do Estado do Amazonas, Escola Superior de Tecnologia, Laboratório de Química Aplicada à Tecnologia, Av. Darcy Vargas, 1200, Manaus, AM, 69050-020, Brazil

**Keywords:** Honey, Stingless bee, *Melipona* sp, *Apis* sp, Antibacterial activity, Flavonoids, HPLC

## Abstract

**Background:**

Honey has been identified as a potential alternative to the widespread use of antibiotics, which are of significant concern considering the emergence of resistant bacteria. In this context, this study aimed to evaluate the antimicrobial activity of honey samples produced by a stingless bee species and by *Apis* sp. against pathogenic bacteria, as well as to identify the presence of phenolic compounds.

**Methods:**

Honey samples from the stingless bee *M. compressipes manaosensis* were collected twice, during the dry and rainy seasons. Three commercial honey samples from *Apis* sp. were also included in this study. Two different assays were performed to evaluate the antibacterial potential of the honey samples: agar-well diffusion and broth macrodilution. Liquid-liquid extraction was used to assess phenolic compounds from honey. HPLC analysis was performed in order to identify rutin and apigenin on honey samples. Chromatograms were recorded at 340 and 290 nm.

**Results:**

Two honey samples were identified as having the highest antimicrobial activity using the agar diffusion method. Honey produced by *Melipona compressipes manaosensis* inhibited the growth of *Staphylococcus aureus, Escherichia coli* (0157: H7), *Proteus vulgaris, Shigella sonnei and Klebsiella* sp*.* A sample of honey produced by *Apis* sp. also inhibited the growth of *Salmonella paratyphi*. The macrodilution technique presented greater sensitivity for the antibacterial testing, since all honey samples showed activity. Flavonoid rutin was identified in the honey sample produced by the stingless bee.

**Conclusions:**

Honey samples tested in this work showed antibacterial activity against Gram-positive and Gram-negative bacteria. The results reported herein highlight the potential of using honey to control bacterial growth.

## Background

Honey is a natural sweetener available all over the world [1]. The antimicrobial potential of this natural product was first described a century ago. However, only recently, this knowledge has been submitted to strict scientific evaluation.

Cooper et al. [2], carried out a study on infected wounds, where the main goal was to extend the current limited knowledge of pathogen susceptibility to exposure to honey, along with the evaluation of the efficacy of honey against resistant organisms, in order to explore its mechanism of action. The authors used 18 strains of methicillin-resistant *Staphylococcus aureus* (MRSA), and seven strains of vancomycin-sensitive *Enterococcus faecalis* isolated from infected wounds. All strains were found to be sensitive to manuka and pasture honey samples, in *in vitro* experiments, demonstrating that honey can be used as an effective wound antiseptic, with a broad spectrum of antimicrobial activity.

Nowadays, it is recognized that most types of honey have antibacterial activity and that this activity is dependent on physical and chemical factors. The viscosity of honey is sufficiently high to create a physical barrel that inhibits the contamination of the wound by infectious agents present in the air. Due to its high sugar concentration, honey eliminates most bacteria by osmosis. The antibacterial activity can also be partially attributed to the acidity of honey, the presence of phytochemical components such as flavonoids and phthalic acids and, most importantly, the action of oxygen peroxide, produced in honey due to the presence of the glucose oxidase enzyme secreted by the hypopharyngeal glands of honeybees [3].

Osmosis and hydrogen peroxide have long been considered as the main factors responsible for the antibacterial activity of honey [4]. However, the verification of non-peroxide antibacterial activity in honey diluted to low concentrations has brought attention to the presence of other antibacterial agents [5].

Among the chemical components in honey which could be responsible for the antibacterial activity, flavonoids and phenolic acids are the most studied. One reason for such interest is that these molecules present innumerous types of biological activity, including antibacterial properties [6]. Several researchers have verified the antibacterial activity of flavonoids isolated from honey and prominent results have been reported for manuka honey from New Zealand. The authors found that methyl syringate is the major constituent of the phenolic fraction of manuka honey (approximately 70% w/w), which presented antibacterial activity [7]. This activity is probably due to the ability of flavonoids to form complexes with soluble proteins and with the bacteria cell wall [6].

In the past few years an increase in the number of research groups dedicated to studying the antibacterial activity of honey can be noted, which has promoted the publication of several papers regarding this activity and verifying its efficiency. These findings have also promoted the interest of companies dedicated to the commercialization of the high level of antibacterial activity of honey, which have provided financial support for research in this area, especially concerning clinic assays.

Currently, around 20,000 honeybee species in habit the most diverse ecosystems around the world. Bees from the subtribe Meliponina are known as indigenous stingless honeybees, and the genus *Melipona Illiger*, 1806, has a high number of species distributed along the neotropical region, with greatest diversification in the Amazon [8]. The honey produced by these bees is considered exotic, with a characteristic flavor and aspect. For this reason it has become a product with high market demand, achieving higher prices than the honey produced by bees of the *Apis* genus, commercialized in different regions of Brazil. In spite of the existence of extensive literature regarding different aspects of the Brazilian stingless honeybee biology [9], there are still only a few studies which have addressed the physico-chemical characteristics and pharmacological properties of its honey, required to define quality standards for its commercialization.

The determination of the antimicrobial potential of the honey from Amazonian stingless bees could identify this honey as an attractive low cost alternative for treating bacterial infections, along with the possibility of promoting a production chain for these native bee products. In this context, the aim of this study was to investigate the antibacterial activity and flavonoid profile of honey produced by the native stingless bees *Melipona compressipes manaosensis*.

## Methods

### Honey samples

Honey samples from the stingless bee *M. compressipes manaosensis* (Apidae, Meliponinae) were provided by the Research Group on Bees (GPA) from the National Institute of Amazon Research (INPA) in Manaus, Amazonas (honey A). Honey samples were collected twice, during the dry and rainy seasons. The harvesting took place in March 2009 (dry season) and November 2009 (rainy season). It was collected 3 samples from each season.

These *Melipona* species keep the honey in compartments within the hives, referred to as pots, and for this reason an adapted 20 mL syringe containing a sterile tube was used to withdraw the honey from the pots. This procedure was carried out by trained GPA technicians. The honey withdrawn was immediately stored in sterile dark glass recipients to avoid photodegradation, and kept at 5°C.

Three commercial honey samples were also included in this study: eucalyptus honey (predominant blooming) from Rio Grande do Sul (honey B); honey produced by *Apis* sp. bees from Ceará (honey C); and honey produced by the stingless bees known as Jandaíra (*M. subnitida*, Apidae, Meliponinae) produced by beekeepers from the community of the Tupé Sustainable Development Reserve (RDS Tupé), located in the rural zone of Manaus (honey D). Harvesting of honey B-D took place in 2009.

### Bacterial strains

Strains of *Staphylococcus aureus*, *Escherichia coli* (O157:H7), *Proteus vulgaris, Shigella sonnei, Salmonella paratyphi and Klebsiella* sp. were kindly provided by the Tropical Virology Laboratory of INPA. Bacterial cultures were kept in nutrient agar at 36°C.

The bacterial strains were previously tested regarding its antibiotic susceptibility, as showed in Table 1.

**Table 1:** Antibiotic susceptibility presented by different bacterial strains used in this work

**Bacterial species**	**Antibiotic**
*Staphylococcus aureus*	VAN (MIC < 4 mg/L)
*Escherichia coli*	CHL / CAR (MIC < 4 mg/L)
*Shigella sonnei*	STR (MIC < 4 mg/L)
*Salmonella paratyphi*	CHL / CAR (MIC < 4 mg/L)
*Klebsiella* sp*.*	GEN (MIC < 4 mg/L)
*Proteus vulgaris*	CAR / STR (MIC < 4 mg/L)

*VAN* - vancomycin; *CHL* - chloramphenicol; *CAR* - carbenicilin; *STR* - streptomycin; *GEN* - gentamicin; *MIC* - minimum inhibitory concentration.

### Antibacterial activity assays

For the antimicrobial assays the bacterial strains were grown overnight in Müeller-Hilton broth at 36.5°C. The bacterial concentration was standardized to 3 × 10^8^ CFU ml^-1^ using the McFarland scale.

In this study two different assays were performed to evaluate the antibacterial potential of the honey samples: agar-well diffusion and broth macrodilution.

For the well diffusion assay Müeller-Hilton solid media was prepared on petri dishes, followed by autoclaving at 121°C for 20 min. Approximately 5 ml of the sterilized media was poured onto the dishes (65 × 15 mm), in an aseptic environment. The diffusion test was performed according to Hernández et al. [10], with a few modifications. Seven wells of 3 mm were made in the agarized medium after inoculation with the bacterial strains. Honey samples were diluted in bidistilled water in the proportions of 1:1, 1:2, 1:4, and 1:8 (v/v). The wells were filled with 30 μl of the honey samples (diluted samples, non-diluted samples and bidistilled water as the negative control). The plates were incubated at 36.5°C for 24 h before visual assessment of the inhibition zones. The experiment was repeated three times. The quantification of microbial growth inhibition was determined by measuring the diameter of clear zones of microbial growth around the wells in the agar (including the well itself). The minimum inhibitory concentration (MIC) was considered the lowest concentration capable of inhibiting the visible bacterial growth.

For the bacterial broth macrodilution assay, appropriate amounts of the honey samples and the culture broth to give the desired concentrations (1:1, 1:2, 1:4, 1:6 and 1:8; v/v) were placed into 5 ml tubes. The bacterial standardized inoculum (30 μl) was poured into the tubes, homogenized and incubated at 36.5°C for 24 h. Samples taken from the tubes were inoculated into petri dishes containing nutrient agar to verify bacterial growth. Inhibition of bacterial growth was visible as a clear broth and the presence of growth was detected by the presence of turbidity. The positive control was the tube containing the culture broth and the bacterial inoculum; the negative control contained the culture broth and the honey sample. The experiment was repeated three times.

### Minimum bactericidal concentration (MBC) of honey

The MBC is the lowest honey concentration capable of killing a bacterial population. It was determined using the incubated tubes from the macrodilution assay, where a 100 μl sample from each tube was inoculated into petri dishes containing nutrient agar. The plates were incubated at 36.5°C for 24 h. The MBC was considered the lowest concentration of honey where no bacterial growth was observed on the agar surface (99.9% of bacterial death) [11]. All tests were repeated three times.

### Statistical analysis

All of the experimental results were expressed as mean ± standard deviation (SD) of three determinations. The presence of a significant difference between honey samples was determined by one-way ANOVA followed by Tukey test (p ≥ 0.05) comparison using BioStat 5.3.5.

### Phenolic compounds extraction from honey

Liquid-liquid extraction with ethyl acetate was used, according to the methodology described by Wahdan [12]. A 20% honey stock solution was prepared in distilled water, and after separating 50 ml of this solution the pH was adjusted to 3.5. To this 50 ml aliquot, 50 ml of ethyl acetate and 1 g of sodium chloride was added in a separation funnel. The funnel was shaken for 5 min for the extraction of phenolic compounds. The ethyl acetate fraction was then withdrawn and stored in a test tube. This procedure was repeated three times. After the extraction, 150 ml of ethyl acetate honey extract, was obtained and submitted to vacuum evaporation at 30°C. The concentrated extract was stored at -18°C.

### Sample preparation for chromatography

An aliquot (2 ml) of each concentrated sample was collect with a 5 ml sterile syringe and filtered through 0.45 μm Teflon membranes. The filtered samples were stored in sterile 2 ml vials. Prior to the injection into the chromatographer column, 400 μl of the samples were added to 600 μl of previously filtered methanol.

### Preparation of standards

Apigenin (5 mg) and pinocembrin (25 mg) were acquired from Sigma-Aldrich Co. The rutin standard was kindly donated by the Laboratory of phytochemistry of the Federal University of Amazonas (UFAM). Stock solutions were prepared by the dissolution of each compound within HPLC-grade methanol. Apigenin and rutin were diluted in 10 ml of methanol and pinocembrin was dissolved in 50 ml of the solvent. The standard solutions were filtered through 0.45 μm Teflon membranes and injected into the chromatographer column under the same analytical conditions used for the honey samples.

### High performance liquid chromatography (HPLC) analysis

The analysis was performed in a Varian HPLC System equipped with an autosampler. For the separation of phenolic components a reverse-phase C-18 column (150 × 4.6 mm × 1/4) was used. The mobile phase consisted of an isocratic gradient system as described by Ferreres et al. [13] with some modifications. Water and methanol were used as the eluent at a flow rate of 1.00 ml min^-1^. To achieve better separation, a gradient elution was used starting with 30% methanol which remained isocratic for the first 15 min, followed 40% methanol at 20 min, 45% methanol at 30 min, 60% methanol at 50 min and 80% methanol at 52 min before becoming isocratic again until the end of analysis at 60 min. Chromatograms were recorded at 340 and 290 nm.

### Flavonoid identification

The flavonoids were identified by two techniques. Firstly, a chromatographic comparison between the peak retention times obtained for the samples and for the standard flavonoids was carried out. The technique of standard addition was also used, which consists of adding known amounts of each flavonoid standard into the sample. If there is an increase in the peak height, and thus in the peak area, without the appearance of shoulders or distortions, this is strong evidence that this peak is the flavonoid of interest. A comparison of the peak retention times was also carried out.

## Results and discussion

### Agar-well diffusion assays

The results for the antibacterial activity of the honey from the stingless bees *M. compressipes manaosensis* (honey A) collected during the dry and rainy seasons are shown in Table 2. It can be observed that both Gram positive (*S. aureus*) and Gram negative (*E. coli, S. sonnei, P. vulgaris* and *Klebsiella* sp*.*) bacteria were inhibited by the honey collected during the dry season. Many studies have demonstrated the antibacterial activity of honey against Gram positive and Gram negative bacteria, and *S. aureus* and *E. coli* are among the most studied microorganisms [2,14]. However, most of these studies used honey produced by *Apis mellifera* bees, and only a few studies on the antibacterial activity of the honey from Amazon stingless bees have been reported, including the investigation of the influence of tropical seasonality on antibacterial activity [15].

**Table 2:** **Diameter of inhibition zone (mm) produced by honey from the stingless bees *Melipona compressipes manaosensis *collected during dry and rainy seasons for different bacterial strains, using agar-well diffusion assays**

**Collection season**	**Honey dilution**^**c**^	**Inhibition zone of bacterial species**^**a,b**^					
		***S. aureus***	***E. coli***	***P. vulgaris***	***S. sonnei***	***S. paratyphi***	***Klebsiella *sp.**
Rainy	Undiluted	-	15.6^A^ ± 0.1	-	11.2^A^ ± 0.2	-	-
	1:1	-	-	-	-	-	-
	1:2	-	-	-	-	-	-
	1:4	-	-	-	-	-	-
	1:6	-	-	-	-	-	-
	1:8	-	-	-	-	-	-
Dry	Undiluted	18.3^A^ ± 0.4	16.1^B^ ± 0.1	24.1^A^ ± 0.2	12.4^B^ ± 0.4	-	8.2 ± 0.5
	1:1	12.6^B^ ± 0.3	9.2^C^ ± 0.1	20.3^B^ ± 0.5	14.6^C^ ± 0.2	-	-
	1:2	12.1^B^ ± 0.2	-	18.2^C^ ± 0.4	-	-	-
	1:4	-	-	15.6^D^ ± 0.2	12.0^B^ ± 0.1	-	-
	1:6	-	-	13.1^E^ ± 0.3	9.5^D^ ± 0.1	-	-
	1:8	-	-	12.8^E^ ± 0.2	-	-	-

^a^ Values are means ± standard deviations of triplicate assays of three independent experiments.

^b^ Averages with a same letter indicate no significant difference (p < 0.05). ^c^ Diluted in bidistilled water (w/w).

The honey collected during the dry season showed higher activity, inhibiting five out of six microorganisms, at different dilution rates. This honey sample was able to inhibit Gram positive and Gram negative strains. The honey collected during the rainy season, on the other hand, presented lower activity, inhibiting only two microorganisms when undiluted. These results clearly indicate the influence of seasonality over the antibacterial activity of the honey obtained from *M. compressipes manaosensis*.

During the rainy period high honey production is observed due to the elevated level of blooming in the Amazon region, which increases the availability of nectar for the bees. The dry season, with the absence of rain, is considered a period of low honey production since the level of blooming is lower. In this study, the honey collected during the dry season presented a higher antimicrobial activity (Table 2), suggesting that the phytochemical components from the nectar can be associated with the bacterial growth inhibition.

During the season of lower blooming the bees are fed with artificial nectar composed of a mixture of sugars or sugarcane molasses to avoid colony losses, due to the lack of natural food. Therefore, the honey samples collected during the dry season, which presented higher antimicrobial activity, would be expected to have a lower percentage and diversity of phytochemical components from flower nectar. On the other hand, the honey samples collected during the rainy season, which would be richer in these components, exhibited a lower antimicrobial activity, and therefore, the difference in antibacterial activity observed for honey samples from different seasons can be explained by access of the bees to plants that provide nectar richer in bioactive molecules during the dry season or by the plants physiologic response to the region’s dry season. It should also be mentioned that these honey samples are from the same bee species, and from the same bee colonies, collected during two different seasons.

The bees of the *Melipona* species keep the honey in the hives in pots, which are made of a combination of vegetal resin and beeswax [16]. This combination may influence the phytochemical composition of the honey, considering the substances present in plant resins. This, in turn, could influence the antibacterial activity of a certain honey, considering that the plants in the Amazon region respond physiologically to environmental changes, mainly those related to the drying up and flowing of the rivers. This response can lead to the inhibition of the production of some substances during periods of river flow, to favor plant nutrition and growth or to enhance production of defense and maintenance substances during the dry period in order to guarantee survival [17]. These substances can be deposited in the honey through direct contact with the pots produced by the bees. However, the quantities of these substances transferred to honey may be too small to alone provide the antibacterial activity of honey; however, part of the observed activity could be attributed to these substances present in the pots [7]. In this case, entomologic factors could contribute to the difference in the antimicrobial activity, for example, the presence of hydrogen peroxide in honey resulting from the presence of glucose oxidase produced by the bees. Hydrogen peroxide is one of the most important factors associated with antimicrobial activity in honey [18]. Since the presence of hydrogen peroxide was not investigated in this study, it was not possible to verify whether or the activity was, in fact, due to this compound.

It can be noted that some pathogenic microorganisms were inhibited by diluted honey in the proportion of 1:4 (20.0% v/v), 1:6 (14.3% v/v) and 1:8 (11.1% v/v), which are more dilute than the required concentration of 22%, according to Chirife et al. [19]. The minimum concentration of a sugar solution required to prevent the growth of most pathogenic bacteria is 29% (w/v) along with a water activity value (a_w_) of between 0.86 and 0.89. These values for the sugar concentration and a_w_ are equivalent to a 22% honey concentration [4].

The results for the antimicrobial activity of three different honeys produced by the *Apis* sp. bees (sold commercially) are presented in Table 3. It can be observed that some microorganisms are also inhibited by this type of honey and that honey B presented the highest activity, inhibiting the growth of five of the six test organisms.

**Table 3:** **Diameter of inhibition zone (mm) produced by commercial honeys from *Apis *sp. bees for different bacterial strains, using agar-well diffusion assays**

**Commercial honey**	**Honey dilution**^**c**^	**Inhibition zone of bacterial species**^**a,b**^					
		***S. aureus***	***E. coli***	***P. vulgaris***	***S. sonnei***	***S. paratyphi***	***Klebsiella *sp.**
B	Undiluted	-	26.2^A^ ± 0.3	26.3^A^ ± 0.2	18.2^A^ ± 0.4	14.3^A^ ± 0.1	17.2^A^ ± 0.2
	1:1	-	21.8^B^ ± 0.2	25.4^B^ ± 0.4	16.6^B^ ± 0.1	12.2^B^ ± 0.2	12.8^B^ ± 0.3
	1;2	-	-	15.4^C^ ± 0.3	17.5^A^ ± 0.3	11.1^C^ ± 0.1	-
	1:4	-	-	16.8^D^ ± 0.1	10.2^C^ ± 0.3	10.2^D^ ± 0.2	-
	1:6	-	-	15.1^C^ ± 0.2	7.3^D^ ± 0.2	10.4^D^ ± 0.1	-
	1:8	-	-	-	-	-	-
C	Undiluted	-	7.4^C^ ± 0.2	-	10.3^C^ ± 0.1	-	-
	1:1	-	-	-	-	-	-
	1:2	-	-	-	-	-	-
	1:4	-	-	-	-	-	-
	1:6	-	-	-	-	-	-
	1:8	-	-	-	-	-	-
D	Undiluted	8.7 ± 0.5	14.5^D^ ± 0.3	-	-	-	8.7^C^ ± 0.2
	1:1	-	-	-	-	-	-
	1:2	-	-	-	-	-	-
	1:4	-	-	-	-	-	-
	1:6	-	-	-	-	-	-
	1:8	-	-	-	-	-	-

^a^ Values are means ± standard deviations of triplicate assays of three independent experiments. ^b^ Averages with a same letter indicate no significant difference (p < 0.05). ^c^ Diluted in bidistilled water (w/w).

The results presented in Tables 2 and 3 indicate that the antimicrobial activity exhibited cannot be attributed exclusively to the osmotic pressure of sugars present in honey. Considering that the honey from the stingless bees has a lower viscosity and presents a slower crystallization, when compared to the honey produced by *A. mellifera* bees [20], it could be inferred that the solutions prepared with the honey from *M. compressipes manaosensis* could be even more diluted than the solutions prepared with the commercial honeys obtained from *Apis* sp., submitted to the same dilution rates.

It is evident that the osmotic pressure contributes to the action of high sugar concentration solutions, but the results obtained here reveal that the activity attributed to osmosis and exerted by these honeys is not sufficient to inhibit the growth of some bacterial species. Thus, these findings indicate that there are other factors contributing to the observed antimicrobial activity.

Therefore, the results presented herein suggest that this activity can be attributable to components of entomological origin and/or of phytochemical origin, along with the osmotic pressure. Some authors have suggested that the bee species is mainly responsible for the antimicrobial activity [21], while others have verified that the phytogeographic region is the main factor responsible for the differences observed in the antimicrobial activity [15]. However, Bogdanov [22] suggested that both the bees and the plants influence the activity, acting in a synergistic way.

Comparing the results for the activity of the honey samples (Tables 2 and 3), it can be verified that the bacteria *S. aureus* was inhibited only by honey A from the dry season and honey D. Honey A from dry season inhibited *S. aureus* growth when applied undiluted and at two dilution rates (MIC value of 33.3% v/v). The largest inhibition zone was observed when the sample was applied undiluted. *E. coli* was inhibited by all five honey samples, and honey B was the sample that promoted the largest inhibition zone (MIC value of 50.0% v/v). *S. sonnei* was inhibit by four of the five honey samples and honey B was the sample that promoted the largest inhibition zone when compared to the other honey samples tested in this study (MIC value of 14.3% v/v). However, it is worth mentioning that the largest inhibition zone observed when using honey A was at the dilution rate of 1/1. The bacteria *P. vulgaris* was inhibited by three honey samples, and honey B was the sample that leads to the largest inhibition zone (MIC value of 14.3% v/v). *Salmonella paratyphi* was only susceptible to honey B, being inhibited until the dilution rate of 1:6 (MIC value of 14.3% v/v). The bacteria *Klebsiella* sp. was inhibited by three honey samples, and again honey B showed the most promising results (MIC value of 50.0% v/v).

In general, the highest inhibition zones were observed when the samples were applied undiluted, except for honey A against *S. sonnei*, which showed the greatest susceptibly when the honey was used at a dilution rate of 1/1 (50.0% v/v). These results allow us to report that the commercial honey from eucalyptus (honey B) not only inhibited the greatest number of pathogenic microorganisms, but also presented the greatest effect on the tested bacteria, as it was able to prevent bacterial growth at five of the six dilution rates evaluated.

Honey A from *M. compressipes manaosensis* demonstrated a good quality spectrum of antimicrobial activity, being able to inhibit the growth of five microorganisms (*S. aureus, E. coli, S. sonnei, P. vulgaris* and *Klebsiella* sp.). Within the commercial honeys, honey B produced by the *Apis* bee (predominant blooming of eucalyptus) showed the greatest activity, also inhibiting the growth of five microorganisms (*E. coli, S. sonnei, P. vulgaris, S. paratyphi* and *Klebsiella* sp.).

### Bacterial broth macrodilution assays

The antibacterial results obtained from the macrodilution assays for honey samples are presented in Table 4.

**Table 4:** **Antimicrobial results (macrodilution assays) obtained for honey produced by *Melipona *stingless bees and for commercial honeys produced by *Apis *sp. bees for different bacterial strains**

**Honey sample**	**Honey dilution**^**a**^	**Bacterial species**					
		***S. a:ureus***	***E. coli***	***P. vulgaris***	***S. sonnei***	***S. paratyphi***	***Klebsiella *sp.**
AR	11	+	+	-	-	-	-
	1:2	+	+	-	-	-	-
	1:4	+	+	-	-	+	+
	1:6	+	+	+	+	+	+
	1:8	+	+	+	+	+	+
	Positive control	+	+	+	+	+	+
	Negative control	-	-	-	-	-	-
AD	11	-	+	-	+	+	-
	1:2	-	+	-	+	+	-
	1:4	+	+	-	+	+	+
	1:6	+	+	+	+	+	+
	1:8	+	+	+	+	+	+
	Positive control	+	+	+	+	+	+
	Negative control	-	-	-	-	-	-
B	11	-	-	-	-	-	-
	1:2	-	-	-	-	-	-
	1:4	-	+	-	-	-	-
	1:6	+	+	+	-	+	+
	1:8	+	+	+	+	+	+
	Positive control	+	+	+	+	+	+
	Negative control	-	-	-	-	-	-
C	11	-	-	-	-	+	-
	1:2	-	-	-	-	+	-
	1:4	+	+	+	+	+	+
	1:6	+	+	+	+	+	+
	1:8	+	+	+	+	+	+
	Positive control	+	+	+	+	+	+
	Negative control	-	-	-	-	-	-
D	11	-	-	-	-	+	-
	1:2	-	+	-	-	+	-
	1:4	-	+	-	+	+	+
	1:6	-	+	-	+	+	+
	1:8	+	+	-	+	+	+
	Positive control	+	+	+	+	+	+
	Negative control	-	-	-	-	-	-

A: Honey from stingless bee *Melipona compressipes manaosensis* collected during rainy (R) and dry (D) seasons*.* B: Eucalyptus honey from *Apis* sp. bees acquired at a store in Rio Grande do Sul. C: Honey from *Apis* sp. bees acquired at a store in Ceará. D: Commercial honey from the stingless bee Jandaíra (*M. subnitida*), produced by the community of RDS Tupé (rural zone of Manaus).

^a^ Diluted in culture broth (w/w). + Inhibition zone between 10 and 15 mm. - Absence of inhibition zone.

The *in vitro* studies on the inhibition of pathogens that infect wounds confirmed the broad activity spectrum of honey samples, in the case of both the macrodilution method [21] and the agar-well diffusion assay [15,18]. Most antimicrobial tests applied to honey that involve broth dilution employ the microdilution technique [12], which is performed on a smaller scale than macrodilution but using the same dilution criteria adopted herein.

In this study, the assessment of antibacterial activity was performed using two techniques. The results showed remarkable differences in the nature of the antibacterial activity when the two methods were compared. For instance, using the agar-well diffusion method it was observed that the honey A sample collected during the rainy season showed antibacterial activity only when applied undiluted against *E. coli* and *S. sonnei* (Table 2). When using the broth macrodilution method, however, it was observed that this sample of honey A showed inhibition of bacterial growth at lower concentrations and against other strains (Table 4). Through the macrodilution method, the MBCs were verified at dilution rates of 1:4 (20.0%) for *P. vulgaris*, 1:4 (20.0%) for *S. sonnei* and 1:2 (33.3%) for *S. paratyphi* and *Klebsiella* sp. This difference in the results can be observed for other honey samples, such as the honey produced by the stingless bee Jandaíra. Using the agar-well assay, the honey D produced antibacterial activity only when applied undiluted against *S. aureus*, *E. coli* and *Klebsiella* sp. (Table 3). However, applying the broth macrodilution method, the Jandaíra honey presented a MBC at the dilution rate of 1:8 (11.1%) for *P. vulgaris* (Table 4).

The results shown in Table 4 indicate that the bacterial broth macrodilution method presents higher sensitivity compared to the agar-well assay, probably due to a greater mobility of the active molecules in the liquid broth than in the agar. This behavior can be explained by the possibility of honey being composed of substances that do not diffuse properly within the solid media, which could occur due to differences related to the molecule polarity and the agar medium polarity. In the liquid medium, however, this mass transfer barrier is naturally reduced.

Innumerous factors may influence the difference in the antimicrobial activity observed when using the two techniques. According to Silveira et al. [23], it is expected that the diffusion of extracts of natural products which have more hydrophobic characteristics is hindered in agar, a polar compound. Silveira et al. [23] stated that the inhibited diffusion of natural products may also be related to their hydrosolubility and molecular weight.

Considering that the samples analyzed were aqueous honey solutions, and considering the polar characteristics of the agar medium, it can be assumed that the solvent should easily diffuse into the agar. Therefore, there is a greater possibility that the hydrophilic compounds present in honey will diffuse easily, and that the less polar compounds will have inhibited mobility, due to the incompatibility of the polarities. In the macrodilution test, the liquid medium allows good mobility for both polar and non-polar molecules, since there is no agar barrier to inhibit the diffusion of the non-polar molecules, such as flavonoids and phenolics. This may be a determinant factor in terms of the differences observed between the two methods.

### HPLC analysis

**Figure 1:**
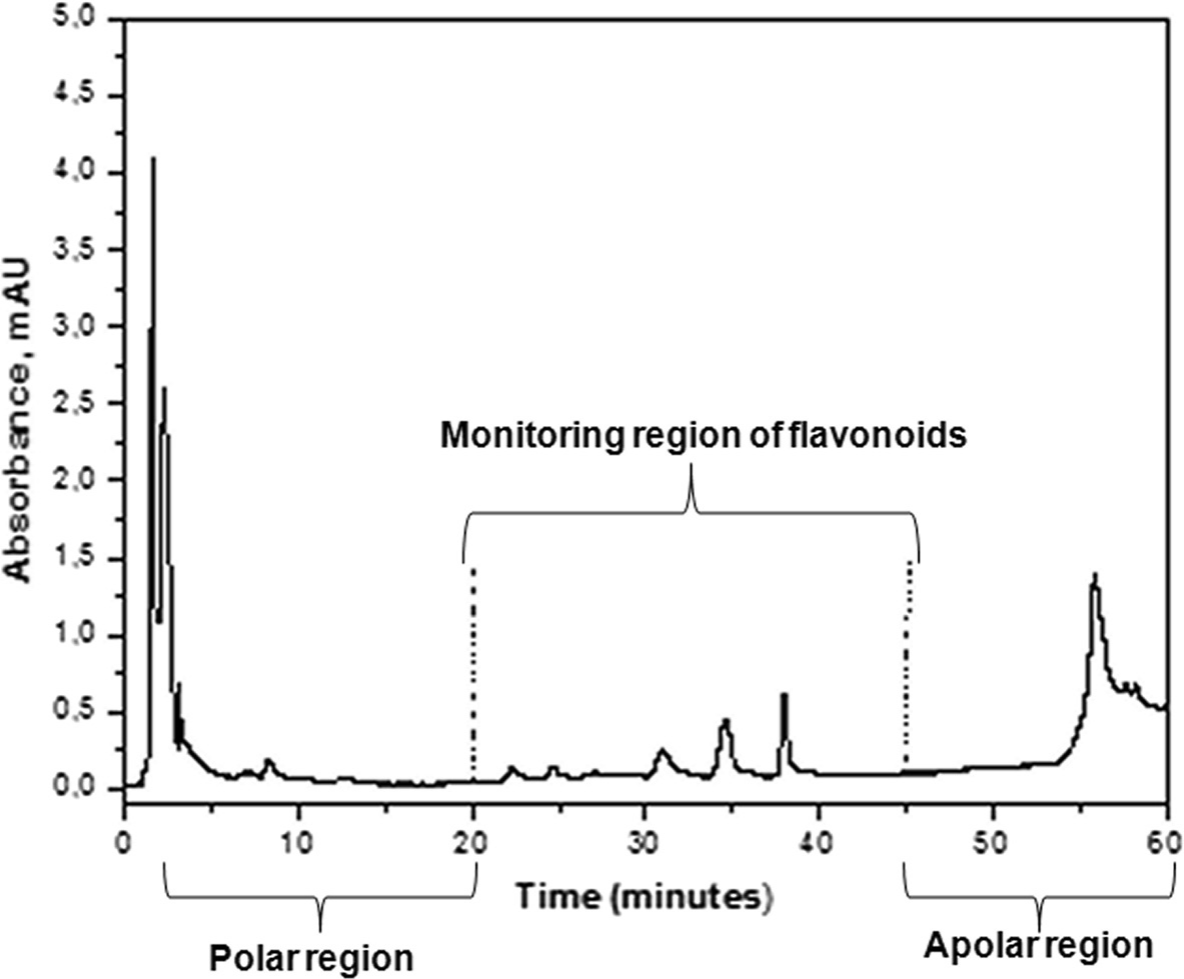
HPLC chromatogram for honey sample showing the monitoring region for flavonoid corresponding peaks (340 nm).

Based on the chromatograms obtained for the flavonoid and phenolic acid standards, along with the analysis methodology employed in this study, it was possible to delimitate the chromatogram regions were the peaks related to the flavonoids and phenolic acids appear. The peaks associated with retention times of between 0 and 10 min correspond to compounds of greater polarity, since the eluent used during the beginning of HPLC analysis has a polar nature. As the time of the chromatographic run increases, the solvent polarity decreases and, therefore, the peaks that appear on the chromatogram correspond to less polar molecules. It is important to consider that the stationary phase used for these analyses was a C18 column. The run began with 30% methanol and this percentage increased over the 60 min run time, reaching 80% by the end of the analysis. Thus, considering this solvent gradient, the more polar compounds were released from the column after shorter retention times, while the less polar compounds were associated with longer retention times. According to Ferreres et al. [13], adopting this technique, peaks that appear between retention times of 20 and 45 min are probably the peaks related to flavonoids and phenolic acids. In this regard, Figure 1 illustrates the chromatogram regions and corresponding polarities for a honey sample used in this study.

Considering that a UV-type HPLC detector was used, it can be affirmed that the detected components have chromophore groups. These components could be derived from phenolic acids or flavonoids. However, other specific analytical methods need to be employed in order to reveal the chemical structures of the different honey samples.

The HPLC operating conditions using a methanol-water gradient have been widely applied by many researchers, allowing the separation of good quality peaks on the chromatogram [1,24]. Nevertheless, other solvents can be adopted according to the specific complexity of each honey type.

**Figure 2:**
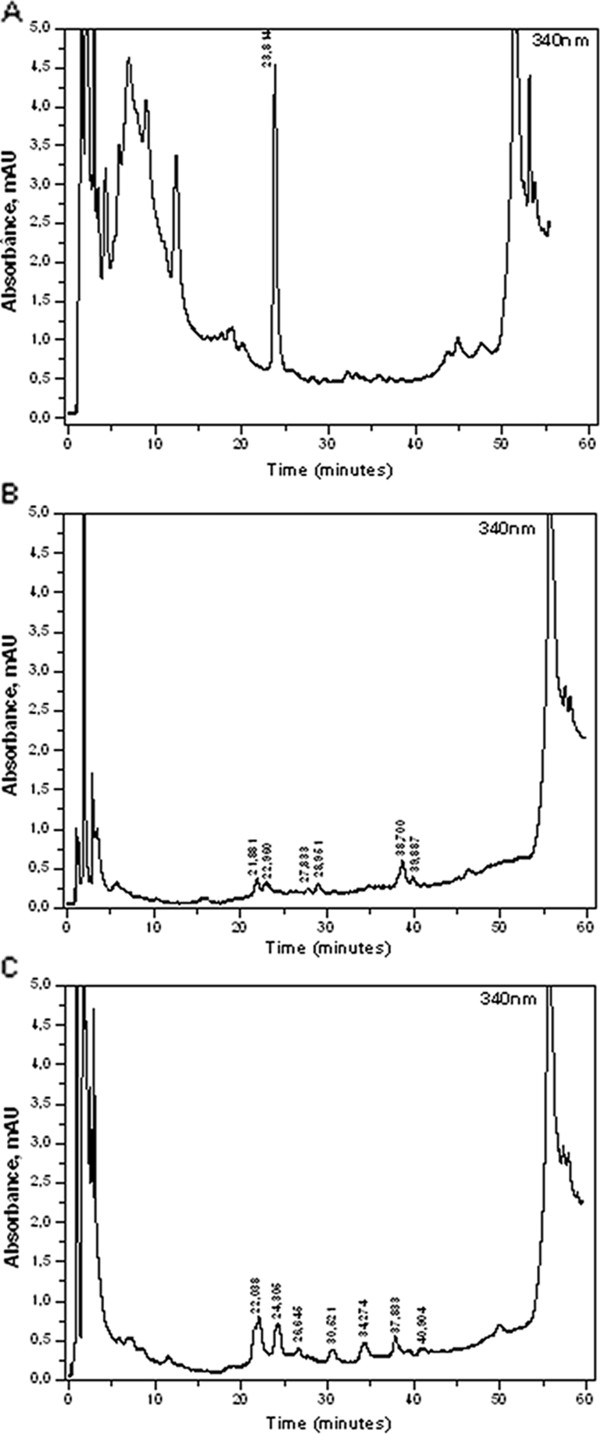
**Phenolic profile of honey samples. (A)** HPLC chromatogram at 340 nm for honey B. **(B)** HPLC chromatogram at 340 nm for honey C. (**C**) HPLC chromatogram at 340 nm for honey D.

Many researchers aiming to identify the phenolic compounds in honey have used aqueous solvents with low pH, usually prepared with formic or acetic acid [7,24]. According to Michalkiewicz et al. [25], this procedure is used due to the acidic nature of most phenolic compounds, which means that an acid mobile phase is required for a satisfactory peak separation, along with a reduced retention time within the column. On the other hand, Bogdanov [22] affirmed that this method is mostly used in HPLC runs that aim to identify phenolic acids in addition to flavonoids. In the present study, water was used without the addition of acids and satisfactory peak separation was obtained in the chromatogram region of interest, where the flavonoids appear (Figure 2).

On analyzing the chromatograms obtained at 290 and 340 nm, and comparing the retention times through the superposition of the observed peaks, it was possible to detect several peaks within the flavonoid monitoring region for all honey samples. However, the flavonoids pinocembrin and apigenin were not identified in any of the samples, indicating that these flavonoids were not present in the honey types used in this study. This result was unexpected, since have been reported the presence of these flavonoids in honey, mainly in honey produced by *Apis* sp bees. Isla et al. [26] found pinocembrin in honeys from the Argentinean Northwest which presented inhibitory effect against *S. aureus*, *E. faecalis*, *E. coli*, *P. aeruginosa*, *K. pneumoniae* and *M. morganii*. Tenore et al. [27] detected pinocembrin in different monofloral honey samples from Italy. Apigenin was found in honey samples from Croatia, according to Kenjeric et al. [28] and in honey samples of different botanical origin from different regions of Sudan, according to Makawi et al. [29].

**Figure 3:**
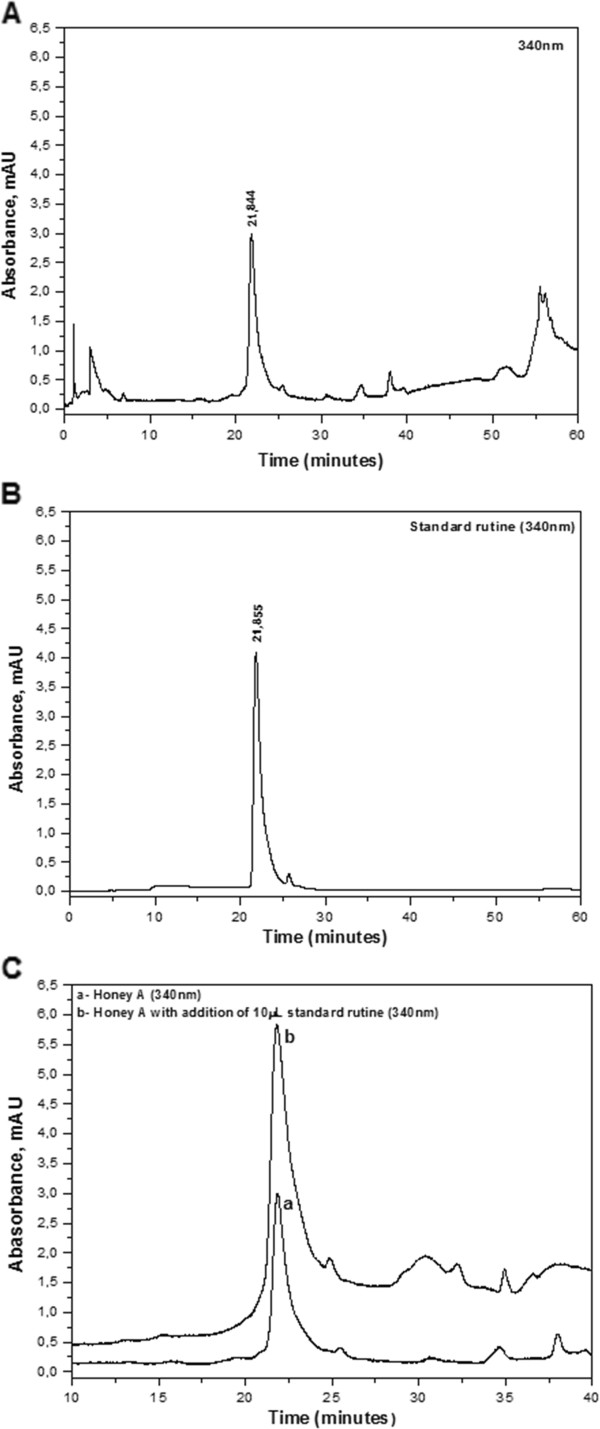
**HPLC analysis of honey samples. (A)** HPLC chromatogram at 340 nm for honey from stingless bees *Melipona compressipes manaosensis*. **(B)** HPLC chromatogram at 340 nm for rutin standard. **(C)** HPLC chromatogram at 340 nm for honey A **(a)** and the same sample after the addition of rutin standard **(b)**.

Nevertheless, a peak for honey A with a retention time of 21.844 min (Figure 3A), which is extremely close to the peak of the rutin standard (retention time 22.855 min), was observed (Figure 3B).

The great similarity between these retention times strongly suggests that this peak corresponds to the flavonoid rutin, which was identified at 340 nm (Figure 3B). Even so, this confirmation requires other analytical techniques to reinforce the molecule identification.

Thus, in order to confirm the hypothesis that the peak with a retention time of 21.844 min corresponded to rutin, the standard addition technique was employed, adding 10 μl of the rutin standard to the honey A sample. The sample with rutine added was injected into the chromatographer column and it was observed that the peak with a retention time of 22.844 min increased, without significant deformations (Figure 3C). This observation suggests that the peak with a retention time of 22.844 min corresponds to the flavonoid rutin. This procedure was repeated three times and the same results were observed.

Rutin was identified in the honey sample with the highest antimicrobial activity. This flavonoid has been previously identified as the responsible for antibacterial activity. The studies performed by Singh et al. [30] showed that rutin from *Pteris vittata* L. exhibited potent activity against *B. cereus*, *P. aeruginosa* and *K. pneumoniae* with the MIC values of 0.03 mg/ml. Basile et al. [31] verified that rutin standard present inhibitory activity against *S. aureus, P. vulgaris, K. pneumoniae, E. cloacae, P. aeruginosa, E. coli, S. typhi* and *E. aerogens* (MICs between 32 and 128 µg/ml). Besides, rutin presents several therapeutic properties associated with the improvement of symptoms related to lymphatic and venous vessels insufficiency, hemorrhagic diseases and hypertension, in addition to antioxidant action [32].

Rutin is a glycosylated flavonoid and belongs to the flavonols subgroup, together with apigenin and pinocembrin, which are found in propolis (or cerumen) produced by *Melipona* bees. Therefore, these flavonoids are not related to the floral origin, since for the production of propolis (or cerumen) these bees use exudates and resins produced by other parts of the plants, rather than the nectar or flower pollen [33,34]. This suggests that transfer of these molecules from the resin to the honey occurs. This transfer is facilitated because the honey of stingless bees is stored in pots built with cerumen. However, factors such as time of contact and temperature must be considered when studying the proportion of glycosylated flavonoids transferred from cerumen to honey, and it should be determined whether the honey age influences the profile of the flavonoid combination.

These observations are in agreement with the results obtained in a study performed with several stingless bee species from Venezuela [34]. Nonetheless, this is the first report of the flavonoid rutin being present in the honey of stingless bees in Brazil.

There are various factors to be considering in exploring the connection between the characteristic flavonoids in the honey of stingless bees and the probable role they play concerning its antibacterial properties. Precise controls must consider the synergistic action of flavonoids and other honey components, due to the complexity of this natural product.

Most of the peaks possibly corresponding to phenolic compounds were observed in the honey samples from stingless bees, indicating a greater variety of floral resources and resins or a richer composition of flavonoids in the botanical source material, when compared to honeys produced by *A. mellifera* bees.

The antimicrobial potential of using phenolic compositions instead of isolated phenolic molecules is widely known. Phenol extracts can be more active than isolated components, since the bioactivity of an individual component can change in the presence of other component within the extract combinations [35], corresponding to a synergic effect [36]. In this study the honey samples were studied without fractioning to determine the antimicrobial activity, since the whole honey sample can be more active than its isolated components due to this possible synergic effect.

Today, the quality of honey and propolis is dependent on the chemical composition and floral origin of the samples. The content of phenolic compounds, such as flavonoids and phenolic acids, is strongly affected by the floral and geographical origin, in addition to the local weather characteristics. For this reason, the identification and quantification of the phenolic compounds present in honey and propolis are of great interest in relation to the development of medicines, since they have proven antimicrobial and antioxidant capacity, which can be attributed to the polyphenols, such as flavonoids and phenolic acids.

## Conclusions

The honey produced by the stingless bee *M. compressipes manaosensis* showed antibacterial activity against Gram-positive and Gram-negative bacteria. The commercial honey obtained from *Apis* sp. bees (eucalyptus honey) showed the highest antibacterial activity. However, it only inhibited the growth of Gram-negative strains.

The macrodilution method was found to be more sensitive than the agar-well diffusion assay for evaluating the antimicrobial activity of honey. This finding indicates that the antibacterial activity may be associated with non-polar compounds, which present a greater resistance to diffusion into the agar.

The flavonoid rutin was identified by HPLC only in the honey produced by the stingless bees *M. compressipes manaosensis.* This is the first report of the presence of this flavonoid in a honey sample produced in Brazil, and the first attempt to obtain a phenolic profile for the honey produced by this stingless bee species. However, several peaks were observed in the chromatogram region considered to be associated with the flavonoid monitoring region, which may be related to other phenolic compounds. The characteristic peaks which appeared in the chromatogram monitoring region of honey samples that presented the highest antimicrobial activity could be related to molecules that are responsible for the pronounced antibacterial activity of these honeys. However, in order to prove this, it would be necessary to separate the fractions for each peak and test them separately.

Our results suggest that there is a possible synergistic action of the different antimicrobial factors associated with honey, such as osmosis, the presence of phenolic compounds and the production of hydrogen peroxide, acting against the pathogenic bacterial strains tested in this study.

## Competing interests

The authors declare that they have no competing interests.

## Authors’ contributions

RBQP performed the sample collection, carried out the antibacterial testing and the HPLC analysis, participated in data analysis and drafted the manuscript. CAC participated in the design of the study, guided the sample collection and antibacterial testing, participated in the analysis and interpretation of data. PMA helped in data analysis and interpretation, performed the statistical analysis and contributed to the review and translation of the manuscript. SDJ conceived of the study, and participated in its design and coordination, carried out the analysis and interpretation of HPLC results and antibacterial testing and critically revised the manuscript. All authors read and approved the final manuscript.

## Authors’ information

RBQP, Masters in Biotechnology, currently a doctoral student at National Institute of Amazon Research, researches on essential oils and other plant secondary metabolites in order to find antifungal activity. CAC, senior researcher at the National Institute of Amazon Research, works in the Laboratory of Virology and Immunology. PMA, Doctor in Organic Chemistry, associate professor at Amazon State University, works with bioprocesses and biocatalysis, along with the prospection of biologically active molecules from Amazon plants and microorganisms. SDJ, Doctor in Physical Chemistry, associate professor at Amazon State University, works with analytical methodologies to assess Amazon biodiversity and performs theoretical studies on biologically active molecules.

## Pre-publication history

The pre-publication history for this paper can be accessed here:

http://www.biomedcentral.com/1472-6882/13/151/prepub
